# Antibacterial Properties of Plasma-Activated Perfluorinated Substrates with Silver Nanoclusters Deposition

**DOI:** 10.3390/nano11010182

**Published:** 2021-01-13

**Authors:** Petr Slepička, Silvie Rimpelová, Nikola Slepičková Kasálková, Dominik Fajstavr, Petr Sajdl, Zdeňka Kolská, Václav Švorčík

**Affiliations:** 1 Department of Solid State Engineering, University of Chemistry and Technology Prague, 166 28 Prague, Czech Republic; nikola.kasalkova@vscht.cz (N.S.K.); dominik.fajstavr@vscht.cz (D.F.) vaclav.svorcik@vscht.cz (V.Š.); 2 Department of Biochemistry and Microbiology, University of Chemistry and Technology Prague, 166 28 Prague, Czech Republic; 3 Department of Power Engineering, University of Chemistry and Technology Prague, 166 28 Prague, Czech Republic; petr.sajdl@vscht.cz; 4 Faculty of Science, J. E. Purkyně University in Ústí nad Labem, 400 96 Ústí nad Labem, Czech Republic; zdenka.kolska@ujep.cz

**Keywords:** fluorinated ethylene propylene, material morphology, nanostructured surface, plasma treatment, poly(L-lactic) acid, polytetrafluoroethylene, silver nanoclusters, antibacterial properties

## Abstract

This article is focused on the evaluation of surface properties of polytetrafluoroethylene (PTFE) nanotextile and a tetrafluoroethylene-perfluoro(alkoxy vinyl ether) (PFA) film and their surface activation with argon plasma treatment followed with silver nanoclusters deposition. Samples were subjected to plasma modification for a different time exposure, silver deposition for different time periods, or their combination. As an alternative approach, the foils were coated with poly-L-lactic acid (PLLA) and silver. The following methods were used to study the surface properties of the polymers: goniometry, atomic force microscopy, and X-ray photoelectron microscopy. By combining the aforementioned methods for material surface modification, substrates with antibacterial properties eliminating the growth of Gram-positive and Gram-negative bacteria were prepared. Studies of antimicrobial activity showed that PTFE plasma-modified samples coated with PLLA and deposited with a thin layer of Ag had a strong antimicrobial effect, which was also observed for the PFA material against the bacterial strain of *S. aureus*. Significant antibacterial effect against *S. aureus*, *Proteus sp.* and *E. coli* has been demonstrated on PTFE nanotextile plasma-treated for 240 s, coated with PLLA, and subsequently sputtered with thin Ag layer.

## 1. Introduction

To date, several polymers have been investigated to determine and improve their antimicrobial activity, and a limited level of biocompatibility has been established in some of them, indicating that the in vivo applications are limited [1]. Although bacterial adhesion can be significantly reduced by material surface coating or application of microstructures, it is difficult to completely prevent the attachment of some bacteria to the surface. Once the bacteria “stick”, a biofilm can form immediately, which is difficult to remove [2]. The mechanism of biofilm formation is a very complex process involving several steps: (i) initial attachment of bacteria to a surface, (ii) maturation, and (iii) dispersion of bacteria attached to the surface [3]. Active surfaces show antimicrobial activity without the release of biocides. They can act with several mechanisms, the so-called spacer effect, at which the biocidal group is attached to the solid surface via a polymer chain [4]. Another strategy of how to fight against unwanted bacterial growth is the binding of metal ions. The limitation of this method is that it significantly reduces the antimicrobial activity of charged agents. Biocide-releasing polymers can be formed either by polymerizing a biocide-releasing backbone or by a composite of a polymer and a biocidal molecule [5]. Covalent immobilization of the surface contributes to the effectiveness of many antimicrobials, which rely on a combination of chemical functionality and spatial conformation, by achieving a specific molecular orientation of the agent on the surface. Therefore, it is possible to preserve the specific structural motifs characteristic for antimicrobials in suspension. However, long-term exposure of polymers to the physiological environment can lead to the loss of chemical and physical antimicrobial surface properties [6]. In the following part of the text, we will focus on different perfluorinated polymers, such as polytetrafluoroethylene (PTFE), tetrafluoroethylene-perfluoro(alkoxy vinyl ether) (PFA), fluorinated ethylene propylene (FEP), or polychlorotrifluoroethylene (PCTFE).

Several biodegradable polymers have been developed as antibiotic carriers for various applications, a hybrid material of chitosan agarose and silver-coated nanocomposite ionogels [7,8], a resorbable composite filler containing antibiotics that exhibits both osteoconductive and antimicrobial properties to reduce the incidence of orthopedic-related infections. Surface-bound polymers exhibit direct antimicrobial activity on the polymer surface [5]. The growing incidence of antibiotic-resistant bacteria is leading to an increased interest in alternative therapeutic concepts and agents [6]. Perfluorinated polymers, such as polytetrafluoroethylene (PTFE) or fluorinated ethylene propylene (FEP), have been widely used previously either activated as cell carriers [9,10,11] or as substrates for subsequent biopolymer superstructure preparation [12,13,14,15]. Many methods have been used to improve the surface properties of PTFE, such as plasma modification, UV-irradiation, application of metal nanostructures, etc. [16]. Plasma modification of perfluorinated substrates led to a change in surface chemistry and the introduction of oxygen groups on the surface of the activated substrate [17]. Good anticorrosive, antiadhesive and antimicrobial properties have been demonstrated by nanocomposites of titanium dioxide and polytetrafluoroethylene (TiO_2_-PTFE) [18]. Silver nanostructures on the PTFE surface were also investigated to study antimicrobial activity. The incorporated silver nanostructures in the PTFE surface showed great antibacterial activity against *E. coli* demonstrated by 99.9% growth reduction after 24 h [19], which does not hold for uncoated PTFE samples [20]. Similar to PTFE, antibacterial properties of activated perfluorooxy alkanes [21], such as PFA [22], were also studied, for which changes in hydrophilic to hydrophobic properties during the grafting process play an important role [23].

Another polymer which has recently received more attention is polyvinylidene fluoride (PVDF). PVDF is a membrane material having excellent properties of high mechanical strength, thermal stability, chemical resistance, and high hydrophobicity when compared to other commercially available polymeric materials, such as polyethylene naphthalate (PEN), polystyrene (PS), polyethylene (PE), or polyethylene terephthalate (PET) [24,25,26]. PVDF membranes have been widely applied in ultrafiltration and microfiltration processes for general separation purposes and are currently being investigated as potential candidates for use in membrane distillation and membrane contractors [27]. Polyphenolic compounds, such as tannin, are potential candidates for the development of PVDF surface coating processes [28]. The conversion of the hydrophobic character of PVDF to hydrophilic was achieved by electron beam grafting and subsequent sulfonation. Increased ion exchange capacity and a lower contact angle of modified PVDF have shown that hydrophilicity played a role in determining membrane contamination [29].

Similar properties as other fluoropolymers can be found for polychlorotrifluoroethylene (PCTFE) but, unlike PTFE, FEP, or PFA, PCTFE, is less deformable and gas permeable which is utilized in gas separations [30]. Moreover, due to its high fluorine content, PCTFE is quite resistant to most chemicals, and also, it is not oxidizable [31]. Many methods are used to improve the PCTFE surface properties, especially non-thermal plasma modification. It was developed using nonthermal plasma (NTP) at the atmospheric pressure, followed by graft polymerization of the hydrophilic monomer [32], and small contact angles for fluorocarbon polymer films were obtained for the PCTFE film [33]. The study proposed a useful range of PCTFE concentrations in which the host properties of poly(methyl methacrylate) (PMMA) can be modified to optimize optical and structural properties without much PCTFE degradation [34]. Most fluorocarbon polymers have a semi-crystalline structure, low glass transition temperatures, and high melting points [35]. The use of these materials for biomedical applications is based primarily on PTFE, which is a fully fluorinated homopolymer that is highly non-reactive and non-toxic when implanted into biological tissues [36]. PVDF-based polymers have low acoustic impedance and a flexible constitution. The possibility of obtaining multi-fiber PVDF yarns was demonstrated, which can be used as biocompatible threads, tubular tubes for vascular prostheses, and multifunctional touch sensors in invasive surgery [35,37]. Owing to their pyroelectric and piezoelectric properties, PVDF-based prostheses are suitable for temperature measurements and the determination of changes in pressure. Fluorine-containing polymers, such as PVDF copolymers, PTFE, and fluoroelastomers, can also be used to make catheters [38] and smart materials for advanced biomedical applications [39]. Clinical and experimental studies have shown that the porosity of PTFE helps the tissue to invade the environment, as well as induces good adhesion and stability of the implant at the host site. However, this material can be considered poorly biocompatible, especially in diabetes, due to moderate infiltration of mononuclear inflammatory cells, such as lymphocytes, macrophages, and foreign body types of multinucleated giant cells [40,41]. PTFE has also been investigated in a three-dimensional expanded form (ePTFE) in combination with poly(lactic-glycolic) acid (PLGA) for use in tissue engineering [42].

Today’s smart materials have found their application in drug delivery. New strategies and technologies enable targeted drug delivery, reduce the incidence of side effects, and improve target selectivity [43]. Functional polymers offer an effective platform for achieving specific targeted drug delivery due to their ability to function as an intelligent system that can sense pathophysiological conditions and promptly react to it [37]. Fluoropolymers can self-assemble into nanomicelles or nanoaggregates in aqueous solutions due to their fluorophilic effect; therefore, hydrophilic or hydrophobic drugs can be encapsulated [44].

In this article, we focused on the study of antibacterial properties of modified and activated PTFE nanotextile and PFA films. Previously published papers aimed on fluoropolymers on foil base, such as PTFE [36] or other fluorine based polymeric substrates [35,36,37,38]. Expanded PTFE was studied in Reference [42]. The first aspect of novelty of this paper is based on application of PTFE in the form of non-woven fabric, adhesive, and elastic material. The combination with not only Ag sputtering, but also immersion into poly-L-lactic acid (PLLA) solution and improved phase separation of PLLA, is also an innovative technique. As a primary modification technique, we applied surface plasma treatment followed by an analysis of the physico-chemical surface properties. Based on the acquired data, we proceeded with subsequent PLLA coating based on immersion of perfluorinated substrates into PLLA solution and/or silver nanocluster deposition. We succeeded in combining the aforementioned procedures so that, for a selected combination, we gained a material with outstanding antibacterial properties against both Gram-positive and Gram-negative bacterial strains, which, to the best of our knowledge, was presented for the first time.

## 2. Materials and Methods

### 2.1. Materials and Chemicals

The experiments were performed using two substrates: PTFE in a form of a nanotextile (Goodfellow Ltd. Huntingdon, Great Britain, product number FP301252) and a tetrafluoroethylene-perfluoro (alkoxy vinyl ether) copolymer (PFA) in a form of a film from GoodFellow (product number FV321050). The PTFE porous membrane properties were: density of 2.2 g cm^−3^, the melting point of 327 °C, the thickness of 45 µm, and pore size of 0.2 µm. PFA is a material that is widely used in situations requiring clarity, flexibility, and continuous operation at higher temperatures. In the performed experiments. PFA in the form of a transparent foil with the following properties was used: density 2.15 g cm^−3^, melting point 315 °C, thickness 52 µm, transparent color.

Polylactic acid (PLLA) (Goodfellow Ltd., M_w_ 20,000) was used as a supplement to study the antimicrobial activity of PTFE samples. PTFE samples were soaked into a solution of 2 g of PLLA dissolved in a mixture of chloroform and methanol in a ratio of 85:15 (*v/v*). Chloroform (CHCl_3_, stabilized with 1% ethanol A.G., M_r_ = 119.38, supplied by Penta, Prague, Czech Republic) and methanol (MeOH, for HPLC, M_r_ = 32.04, supplied by Penta, Czech Republic) were used as solvents. The immersion of the samples took 2 s. After withdrawing the sample from the solvent solution, it was rapidly evaporated and the PTFE surface was coated on both sides with PLLA. PLLA was chosen mainly due to its excellent biodegradability to improve the surface properties of PTFE [45]. The basic properties of the used source PLLA foil were: density of 1.25 g cm^−3^; the glass transition point of 59 °C; thickness of 50 µm; transparent color.

### 2.2. Activation of Substrates

The surface of PTFE and PFA substrates was activated by argon plasma discharge using a Balzers SCD 050 device (Baltec AG, Balzers, Liechtenstein). The conditions during activation were the following: gas pressure 8 Pa, room temperature (RT), the input power of 3 and 8 W, exposure time of 60, 120, 240, and 480 s.

Using the sputtering method, Ag layers of various thicknesses were formed on PTFE and PFA samples with deposition times from 50 s to 300 s. Quorum Q300T ES system with the Pfeiffer vacuum system was applied with the following deposition parameters: table temperature of 20 °C; the pressure of 1 Pa; current of 30 mA. For silver sputtering, targets from Safina a.s. were used (purity of 99.999%). The thickness of Ag layer was determined from weighing analysis with a Mettler Toledo UMX2 microbalance (Mettler-Toledo, LLC, Columbus, OH, USA). The thickness of the deposited layer was calculated from the measured change in weight of 4 samples before and after the deposition. The depolarization high frequency gate was used to discharge the sample surface in order to minimize the influence of surface electrostatic charge on the measurement. After deposition, the determination the deposited Ag thickness was calculated from the weight of the deposited layer and area of the sample and the Ag density.

### 2.3. Characterization of Substrates

The contact angle, characterizing material surface wettability, was measured by goniometry using the Surface Energy Evaluation System (Advex Instruments, Brno, Czech Republic). The measurements of the water drop contact angle (error ±5%) were performed using distilled water on at least 3 samples, at 20 different positions on the material surface at RT.

The surface morphology, roughness, and area of the modified polymers were determined by atomic force microscopy (AFM) using Dimension ICON (Bruker Corp., Billerica, Massachusetts, USA). The samples were analyzed in Scan-Assyst mode using nitride lever SCANASYST-AIR with Si tip (spring constant of 0.4 N.m^−1^). NanoScope Analysis software was applied for data processing. R_a_ (average roughness) is introduced with the surface morphology images.

The elemental composition on the material surface was analyzed by X-ray photoelectron spectroscopy (XPS) using spectrometer ESCAProbeP (Omicron Nanotechnology, Scienta Omicron, Uppsala, Sweden). Atomic concentrations of elements were determined from the individual peak areas using CasaXPS software. The spectra were measured with polymer analysis by XPS, which means using relatively low power of X-ray source (75 W), using monochromatic X-ray radiation (1486.7 eV), and using very low energy of charge compensating electrons (typically 2 eV), all of which protect the measured polymer surface against changes caused by radiation. The data was processed by CasaXPS program, and the measured spectra were compared with our other results and reference analysis and from the NIST database. Peak fitting was based on possibilities of CasaXPS program, background removal was applied by Shirley curve, which allows us to respect the asymmetry of analyzed peaks.

### 2.4. Antibacterial Activity

Antimicrobial tests were performed for two polymers PTFE and PFA. PTFE nanotextile was also coated with PLLA, as described before. All graphs are introduced as proportional to control, which represents 100% of colony forming units (CFU). A set of samples was created to study the PTFE antimicrobial activity:

- pristine PTFE,
- PTFE + Ag 80 s,
- PTFE + Ag 300 s,
- PTFE + plasma 8 W 20 s,
- PTFE + plasma 8 W 240 s,
- PTFE + plasma 8 W 240 s + Ag 80 s,
- PTFE + plasma 8 W 240 s + Ag 300 s,
- PTFE + PLLA,
- PTFE + plasma 8 W 240 s + PLLA,
- PTFE + PLLA + Ag 50 s,
- PTFE + plasma 8 W 240 s + PLLA + Ag 50 s,
- control-physiological solution.

The antimicrobial properties of plasma treated, silver coated or PLLA-coated PTFE samples were monitored for following bacterial strains: Gram-positive *S. aureus* (CCM 3953) and *S. epidermidis* (DBM3179), and Gram-negative *E. coli* (DMB 3138) and *Proteus* sp. (CCM 1799). The bacteria were inoculated from agar plates into Luria-Bertani (LB) medium and then cultured overnight at 37 °C in an orbital shaker. The optical densities of the bacterial cultures were measured at 600 nm and serially diluted. In the amount of 2.10^−4^ of CFU of *E. coli* and *Proteus* sp., 4.10^−4^ of CFU of *S. aureus* and *S. epidermidis* were inoculated per 1 mL of sterile physiological solution (0.9% NaCl) into which the test samples were immersed. After that, the samples were very gently mixed and dynamically incubated at 24 °C for 1, 4, and 24 h. Then, the samples were gently mixed again and 20 μL drops of each sample (3 replicates) were pipetted onto LB agar plates (*E. coli*, *Proteus* sp.) and plate count agar plates (*S. aureus*, *S. epidermidis*). The plates were then incubated at 37 °C for 16 h, after which the number of CFUs was counted and compared with the number of CFU on control plates (bacteria incubated only on a physiological solution at the same time points). The experiments were performed in sterile conditions.

Antibacterial tests for PFA were performed according to the same experimental procedure as in the case of PTFE + PLLA, but only for two representative types of bacterial strains: *S. aureus* (CCM 3953) and *E. coli* (DMB 3138). The following samples were prepared:

- pristine PFA,
- PFA + Ag 60 s,
- PFA + Ag 240 s,
- PFA + plasma 3 W 240 s,
- PFA + plasma 3 W 240 s + Ag 60 s,
- PFA + plasma 3 W 240 s + Ag 240 s,
- PFA + plasma 8 W 240 s,
- PFA + plasma 8 W 240 s + Ag 60 s,
- PFA + plasma 8 W 240 s + Ag 240 s,
- control-physiological solution.

For the sake of clarity, we would like to repeat the information from the previous part of the Experimental section that plasma treatment was represented by the two exposure powers of 3 and 8 W and exposure time of 240 s. Sputtering of Ag nanostructures is described by the sputtering time, which was 60 and 240 s. PLLA represents the immersion of samples into the PLLA solution.

## 3. Results

### 3.1. PTFE and PFA Surface Wettability

In this study, two fluorinated polymers were used (PTFE and PFA). To tailor their surface wettability, they were first subjected to argon plasma treatment at the power of 3 and 8 W for 60, 120, 240, and 480 s. The samples were studied by goniometric analysis, for which water and glycerol were used as measuring liquids. Changes in wetting contact angles were observed as a function of plasma exposure time.

**Figure 1:**
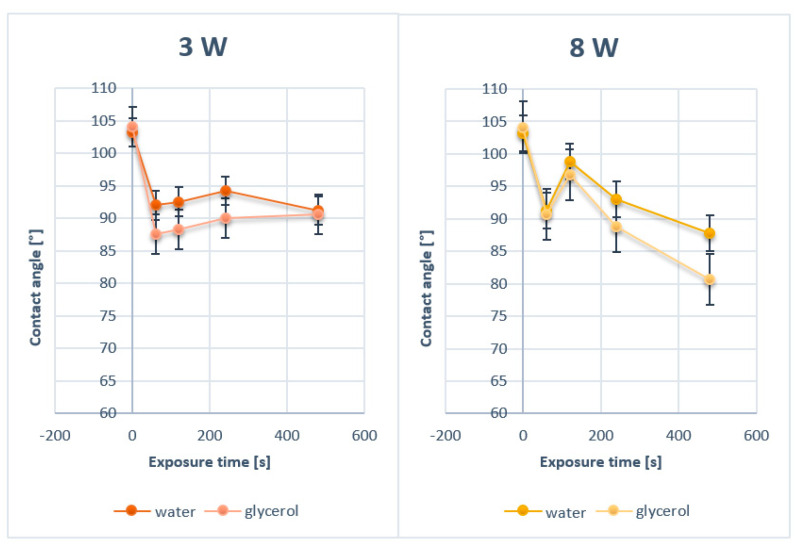
Contact angles of pristine polytetrafluoroethylene (PTFE) and PTFE modified by plasma at 3 and 8 W for 60, 120, 240, and 480 s.

The results of the performed measurements for PTFE are shown in Figure 1. As it is apparent from Figure 1, there was a steep decrease in the surface contact angle of PTFE modified by plasma for 60–120 s. When the length of plasma exposure was prolonged to 120 s, independently of the plasma power, the contact angle increased as a result of which, the PTFE surface wettability decreased again. The sample modified by plasma for 480 s, compared to the unmodified sample, had almost the same or slightly higher contact angle compared with the pristine one. The results show that the plasma modification affects the wettability of the PTFE fabric, but the decrease in the contact angle was much smaller than that of the PTFE film in ref. [9] or other perfluorinated substrates [10]. This difference was quite unexpected and varied from other perfluorinated films. This was probably caused by the specific morphology of these fabric-forming nanofibers, which will be discussed in detail further. The increase in a surface contact angle of PTFE by longer exposures to plasma treatment was caused by the ablation of the already modified surface.

**Figure 2:**
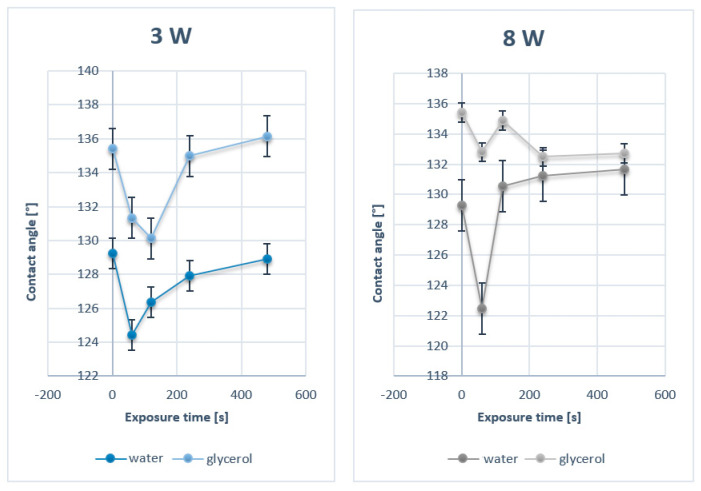
Contact angles of pristine PFA and PFA modified by plasma at 3 and 8 W for 60, 120, 240, and 480 s.

The second substrate, PFA, used in this study was modified by argon plasma at the same conditions as PTFE (3 and 8 W, exposure times of 60, 120, 240, and 480 s). The results of the goniometric measurements for plasma-treated PFA are shown in Figure 2. Similarly to PTFE, an increase in PFA surface wettability was observed for plasma exposure longer than 60 s and a consequent increase in contact angles was apparent with the increasing length of exposure. The changes in contact angle values in Figure 1 and Figure 2 are non-monotonic. This phenomenon is caused by the surface ablation induced by plasma exposure of material. Two major phenomena occur. Firstly, the material is altered by the exposure, which induces surface physico-chemical changes. Secondly, the modified layer is then ablated and removed from the surface, which reveals the bottom layer, which is unmodified. This causes the fluctuations in surface wettability and values of contact angles on studied samples.

### 3.2. PTFE and PFA Surface Morphology

**Figure 3:**
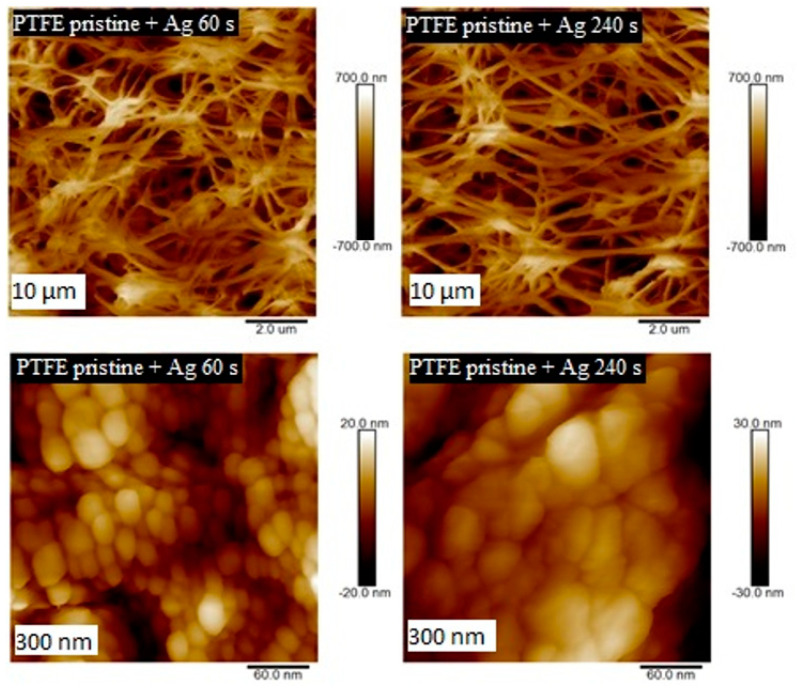
Surface morphology study by AFM for pristine PTFE samples with Ag deposited for 60 and 240 s (scans are of 10 × 10 µm^2^ a 300 × 300 nm^2^).

Two different surface modifications, plasma treatment, and silver sputtering were applied to the studied PTFE and PFA substrates. Both the surface morphology and chemistry before the study of their antibacterial properties were examined on these samples. In Table 1, the results on thicknesses of silver sputtered layers during 60, 120, and 240 s deposition time are summarized for PTFE. Subsequently, we examined the PTFE surface morphology with deposited silver (Figure 3).

**Figure 4:**
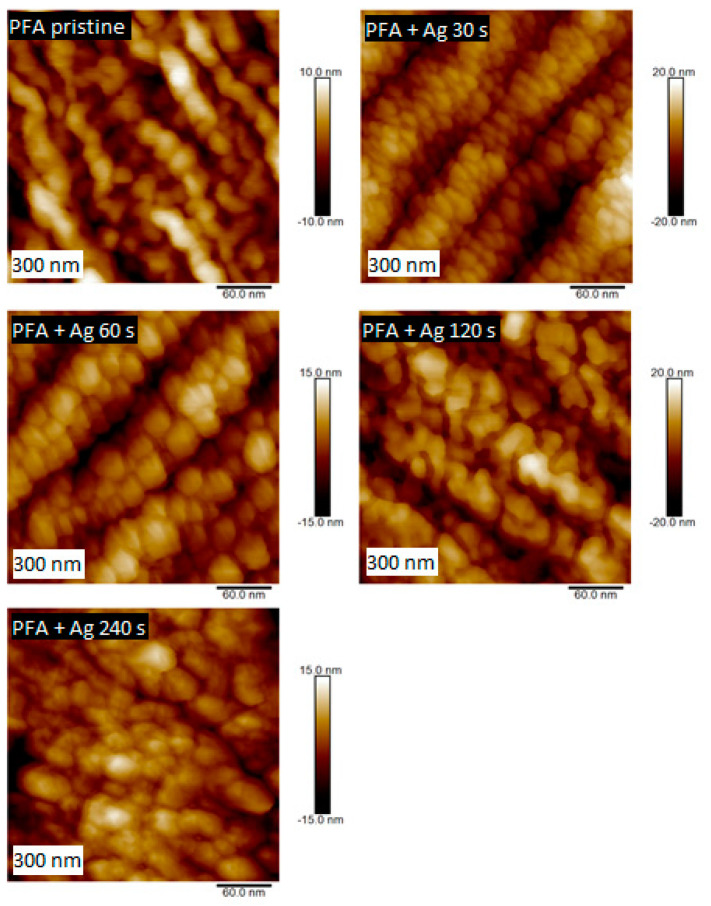
Surface morphology studied by AFM for pristine PFA and PFA deposited with Ag for 30, 60, 120, and 240 s (scan of 300 × 300 nm^2^).

The structures of PTFE and PFA with surface Ag layers, as well as plasma-modified samples, were studied by AFM. Information on the size and shape of arisen surface structures was obtained in the form of 2D and 3D images. The first set of PTFE samples was sputtered with silver with the deposition time of 60 and 240 s. When images with a size of 300 × 300 nm^2^ are compared, it is evident that with growing silver deposition time, the size of silver nanoclusters deposited on PTFE polymer increased (Figure 3). Similar properties were detected also for metalized PFA samples, on which a silver layer was deposited for 30–240 s. When comparing the surface structures of these samples on a 300 nm scan (Figure 4), it is evident, that the deposition time of metal affects the size of forming structures. The longer the deposition time, the greater the surface structures were detected. This effect was confirmed by comparing the surface roughness of PFA samples with sputtered silver. The surface roughness of PFA samples with sputtered silver increased with the growing deposition time of the metal.

**Figure 5:**
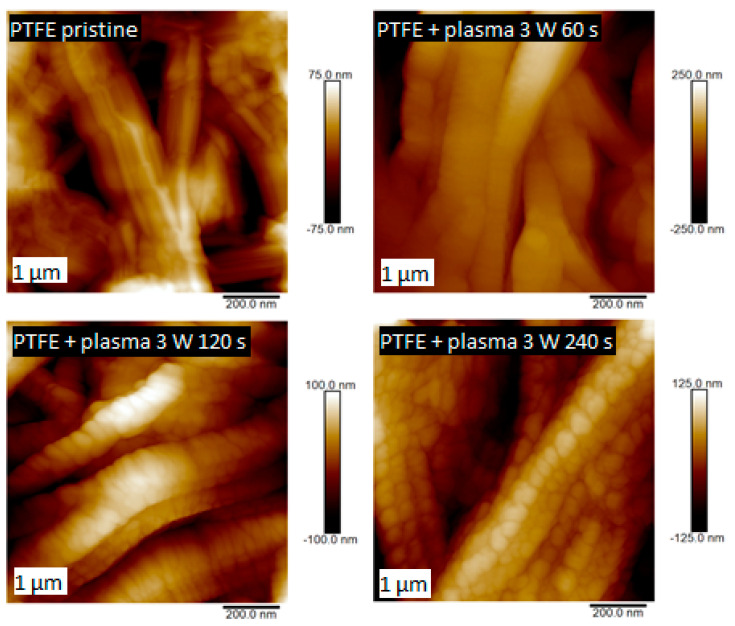
Surface morphology studied by AFM for pristine PTFE and PTFE treated by plasma at 3 W for 60, 120, and 240 s.

The second set of PTFE samples was modified by plasma at 3 and 8 W power outputs and exposure time of 60, 120, and 240 s. The dependence of the shape of the surface structures on the plasma power and exposure time was studied. Significant changes in surface structures were observed already after sample modification with low power of 3 W. In Figure 5, the surface structures of the plasma-modified samples are compared with an unmodified PTFE sample. It is apparent that plasma exposure had a significant effect on the surface structure of PTFE (see Figure 5). The sample modified for 60 s did not show a significant change in surface morphology; however, at longer exposure times, a change in the shape of the surface structures was observed; more specifically, a division into smaller surface nanocluster units occurred.

Next to the PTFE samples, PFA substrates were also studied to determine the effect of argon plasma on the surface structure. Comparison of these two substrates is made based on the similarity of surface chemistry; however, morphologically, they are two different types of materials: a foil (PFA) and nanotextile (PTFE), which will be further noted especially for PFA in AFM analysis.

**Figure 6:**
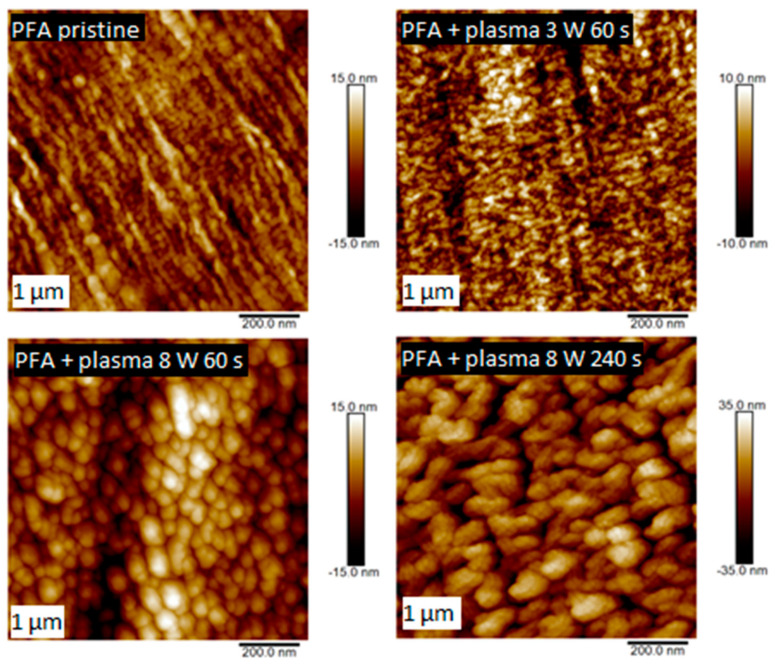
Surface morphology studied by AFM for pristine PFA and PFA treated by plasma at 3 W for 60 s and 8 W for 60 and 240 s.

Comparing the two polymers, it is apparent that the PTFE morphology consisted of long nanofibers with a granular superimposed structure formed by plasma modification. A similar nanostructure appeared on the surface of PFA (Figure 6), but the surface was more homogeneous with different morphology, given in particular the primary shape of the substrate, i.e., a planar foil. PFA samples were compared depending on the plasma exposure time. The surface structures of the plasma-modified PFA samples differed significantly from the unmodified one (see Figure 6).

**Figure 7:**
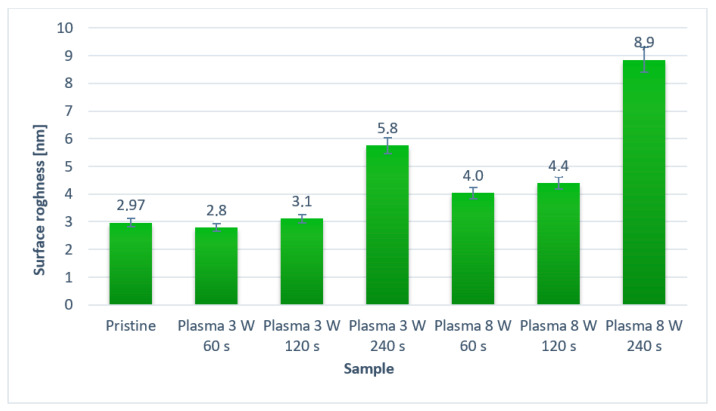
The surface roughness of pristine PFA and plasma-modified PFA by different plasma exposure time and power. A square of 10 × 10 µm^2^ was analyzed.

On the pristine PFA sample (a square of 1 × 1 µm^2^), globular structures are visible, the original fibrous structure was also partially preserved. Plasma modification led to an increase in the number of such nanostructures, an increase in surface roughness and surface fragmentation, as shown in Figure 7. This Figure 7 shows the surface roughness for the untreated PFA and for PFA subjected to plasma treatment at 3 and 8 W for 60, 120, and 240 s. As expected, the PFA surface roughness increased with increasing power and exposure time. A small decrease in PFA surface roughness was detected on the sample modified with the plasma of 3 W for 60 s. This phenomenon is probably caused by the low plasma power and the possibility of partial “smoothing” of the sample in the initial phase of plasma modification. The highest roughness on the PFA surface was detected on a sample treated for the longest exposure time at 8 W power; the roughness was almost three times higher than on the untreated PFA sample.

### 3.3. Surface Chemistry

PTFE and PFA samples modified by plasma at 3 and 8 W and exposure times of 60, 120, and 240 s were studied to XPS analysis. Changes in the surface chemical composition were studied. XPS analysis showed that pristine PTFE samples contained only carbon (C) and fluorine (F) on the surface, while the modified samples additionally contained oxygen (O) and nitrogen (N) at different concentrations depending on the plasma exposure time. The resulting overview spectra for pristine PTFE and plasma-modified samples at 8 W for 240 s are shown in Figure S1. The values of the atomic concentrations of the above elements are shown in Table 2 below.

Since the original PTFE does not contain oxygen or nitrogen, the results showed that the plasma exposure on the samples “bound” oxygen to the fluorine site in the surface layer of the polymer (due to created radicals). As expected, as the plasma modification time rose, the fluorine content gradually decreased and the oxygen content increased. There was also an increase in the atomic carbon concentration but in a much smaller proportion. Despite the binding of oxygen to the surface of plasma-exposed PTFE, no significant decrease in the water contact angle was found (as discussed previously), probably due to the unique nanofiber morphology, which will certainly be the subject of further research.

The chemical composition of the material surface was also studied for pristine and plasma-modified PFA samples. A PFA film, unlike PTFE nanotextile, originally contains oxygen in its structure (although in a relatively low amount); therefore, the modified and pristine sample should differ only in the percentage of the following elements—C, F, and O. The resulting surface atomic element concentrations are shown in Table 3.

As it is apparent from Table 3, with the growing time of plasma exposure, no obvious trend in an increase/decrease in concentrations of individual elements was detected. However, a significant increase in the amount of oxygen depending on the plasma modification was evident. Nitrogen was detected on the surface of most of the plasma-modified samples. This is probably due to its binding from atmospheric nitrogen by a process similar to that of oxygen, i.e. binding to the formed radicals. It is evident that plasma modification leads to a dramatic increase in the oxygen concentration on the PFA surface.

**Figure 8:**
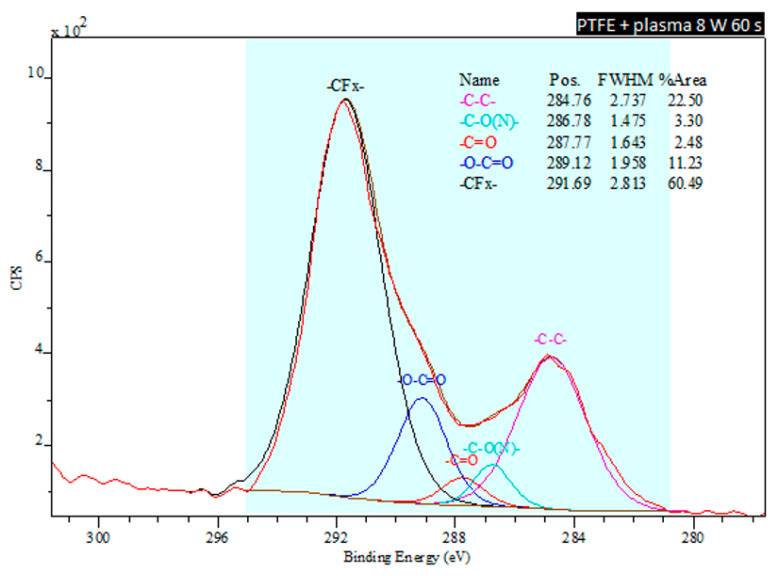
Deconvoluted spectra from an XPS analysis of a plasma-treated PTFE sample exposed to 8 W for 60 s.

**Figure 9:**
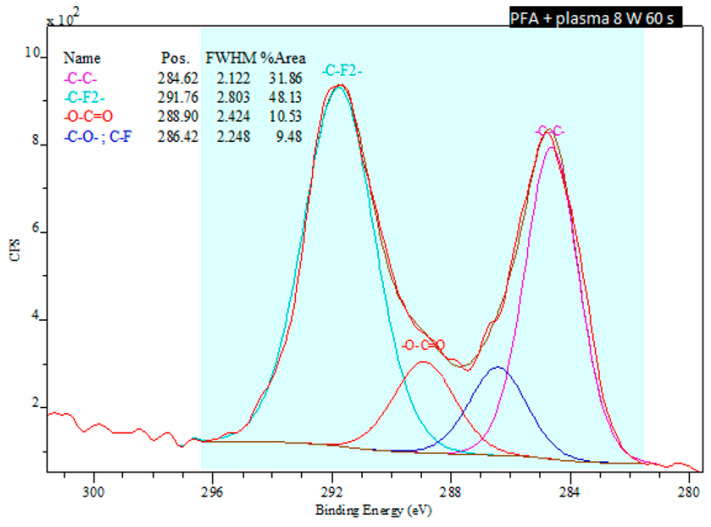
Deconvoluted spectra from an XPS analysis of a plasma-treated PFA sample exposed to 8 W for 60 s.

The XPS method also enables to analyze the specific functional groups on the surface of the aforementioned PTFE and PFA materials and to calculate their concentrations. It was determined that the pristine PTFE sample contains only two types of bonds: carbon-carbon (-C-C-) and carbon-fluorine (-C-F-). In addition to carbon and fluorine elements, PFA material also contains oxygen. Comparing the spectra of both materials at the conditions of plasma modification of 8 W 60 s (Figure 8 and Figure 9) it is apparent that PTFE exposed to plasma has induced a formation of a keto-, carbonyl- and ether- group on its surface. The carbonyl- and ether- groups represent the major groups present on the PFA surface. It is evident that the proportion of the -C-F- group decreases with increasing plasma exposure time for plasma-treated PTFE.

### 3.4. Antibacterial Properties

#### 3.4.1. PTFE

**Figure 10:**
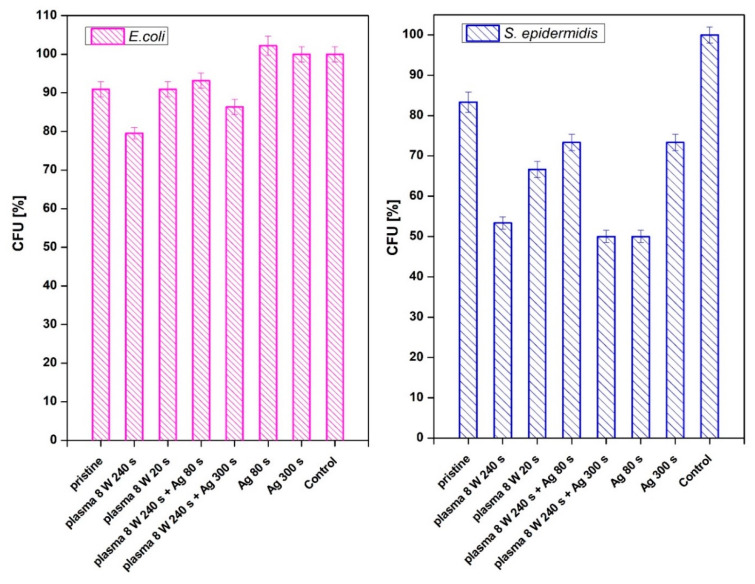
Antimicrobial activity of plasma-activated PTFE further deposited with Ag against bacterial strains of *E. coli* and *S. epidermidis*. Plasma treatment was done at 3 and 8 W for 240 s, Ag deposition for 80 and 300 s. The samples were incubated with the bacterial inocula for 2 h and then grown overnight on agar plates, on which the number of colony-forming units (CFU) was determined.

The antimicrobial activity of PTFE samples was monitored against two bacterial strains: Gram-positive *S. epidermidis* (Figure 10, right column) and Gram-negative *E. coli* (Figure 10, left column). A series of 8 W plasma-modified samples were generated with the plasma exposure of 20 and 240 s and in a combination with Ag sputtered for 80 and 300 s. The results of the material antibacterial activity are reflected by the number of colony-forming units (CFU) of both strains in Figure 10. CFU decreased on each sample in dependence on level of antimicrobial activity.

From Figure 10, it is apparent that both methods of PTFE surface modification (plasma exposure and silver sputtering) affected the antimicrobial activity against *S. epidermidis* significantly, the antibacterial effect for *E. coli* was not observed (Figure 10, left column). As it is obvious from Figure 10, the antibacterial effect was observed on PTFE treated with plasma at 8 W for 240 s or a combination of such plasma treatment with subsequent deposition of Ag for 300 s. A strong antibacterial effect was also observed for sputtering of only a thin Ag layer (time 80 s).

#### 3.4.2. PTFE + PLLA

As a next step, we prepared PTFE samples coated with a PLLA layer and combined with Ag sputtering. The solvent-casting method was applied for the coating of pristine and plasma-treated PTFE. The samples of PTFE nanotextiles (original and plasma-modified) were immersed in a PLLA solution, and selected samples were subsequently deposited with Ag nanolayers. These samples were further studied also for their antimicrobial activity.

**Figure 11:**
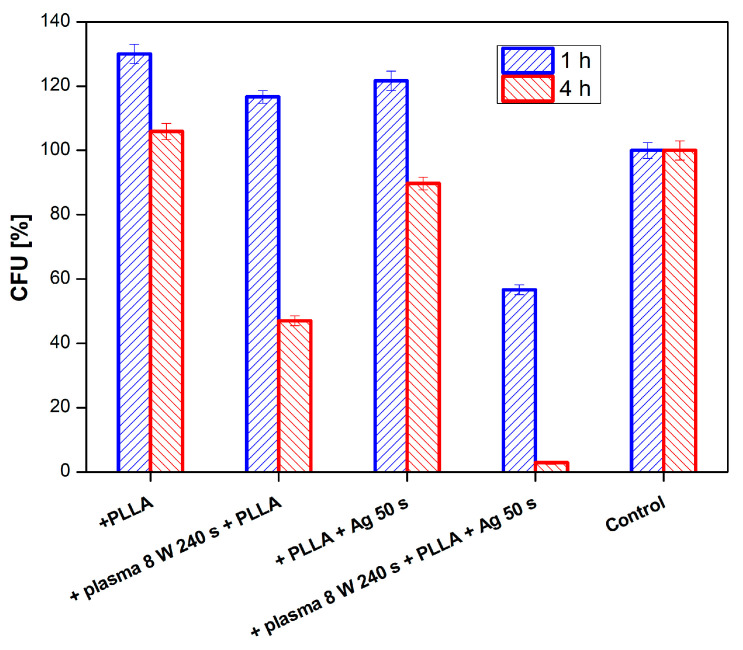
Antimicrobial activity of plasma-activated PTFE + poly-L-lactic acid (PLLA) further deposited with Ag against bacterial strains *E. coli* after 1 and 4 h of incubation. Plasma treatment was done with 8 W for 240 s, Ag deposition for 50 s.

**Figure 12:**
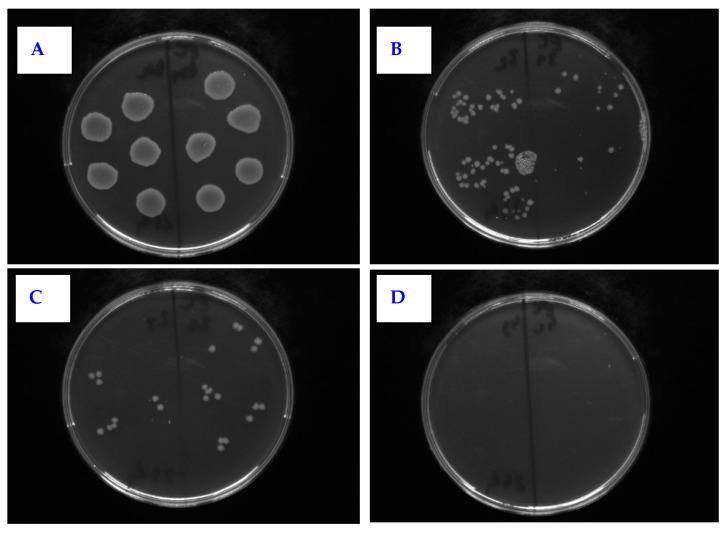
Selected photos of agar plates from antimicrobial tests for PTFE + PLLA against *E. coli.* The samples were the following: (**A**) control sample; (**B**) plasma 8 W 240 s with 2 g PLLA; (**C**) PLLA 2 g + Ag 50 s 20 mA; (**D**) plasma 8 W 240 s PLLA 2 g + Ag 50 s.

The effect of PLLA on the antimicrobial activity of PTFE was studied against four bacterial strains: Gram-positive *S. aureus* and *S. epidermidis* and Gram-negative *E. coli* and *Proteus* sp. In Figure 11, there is shown antimicrobial activity of the tested samples against *E. coli* for PLLA-coated, plasma-modified, and PLLA-coated PTFE samples and both silver-sputtered variants. We would like to mention that the aforementioned physico-chemical properties of PTFE were successfully applied for PLLA coating; however, detailed results of surface morphology after dip-coating are not presented here since we have focused only on the antibacterial properties of the prepared samples. Figure 11 shows that the lowest number of CFU units of *E. coli* after 4 h of incubation was determined for a PTFE sample modified by plasma at 8 W for 240 s, coated with PLLA, and followed by silver sputtering. A significant reduction in the growth of *E. coli* was also achieved by PTFE treated for 8 W for 240 s without silver (Figure 11 and Figure 12).

**Figure 13:**
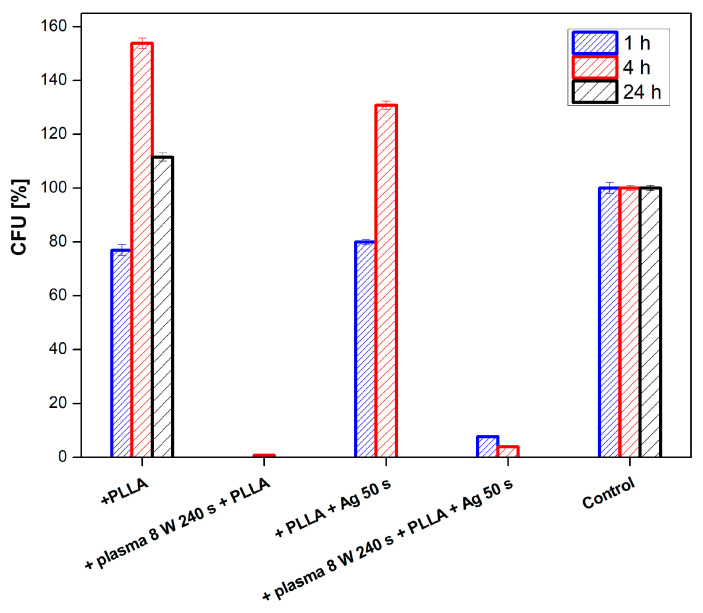
Results of antimicrobial tests for PTFE + PLLA using bacterial strain *Proteus sp.* for samples modified by plasma at 8 W for 240 s, and sputtered with Ag for 50 s.

Even better efficiency of the sample antibacterial activity was achieved against *Proteus sp.* (see Figure 13). The best antimicrobial activity was obtained on plasma-treated samples, subsequently coated with PLLA and sputtered with Ag. Total inhibition of bacterial growth by these samples was achieved already after one hour of incubation and lasted up to 24 h (the longest time period tested).

**Figure 14:**
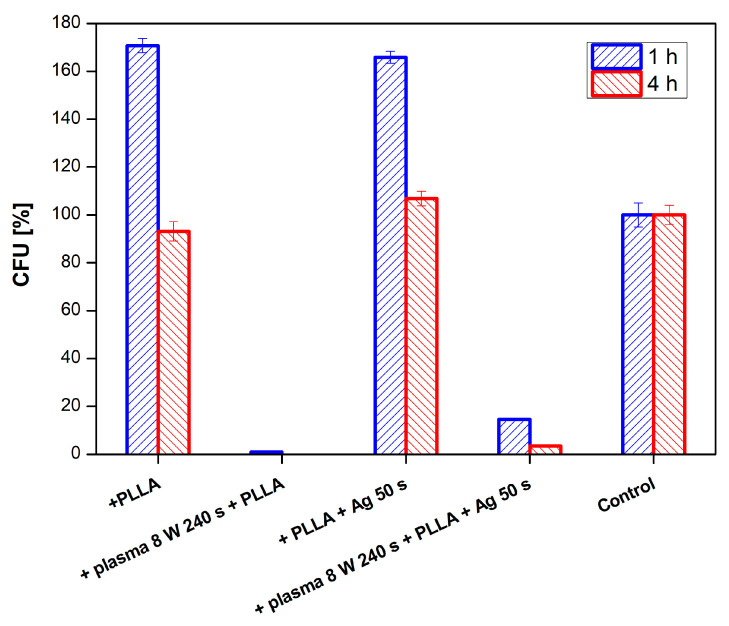
Results of antimicrobial tests for PTFE + PLLA using bacterial strain *S. aureus* for samples modified by plasma at 8 W for 240 s, and sputtered with Ag for 50 s.

The last bacterial strain subjected to antimicrobial tests of PTFE samples was *S. aureus* (Figure 14). After 1 h, there was a growth reduction of *S. aureus* by a PTFE sample treated by 8 W plasma for 240 s with PLLA at both 1 and 4 h of incubation. The same sample further sputtered with silver significantly reduced the number of *S. aureus* CFU after 1 and 4 h of incubation, in comparison to control. The performed tests showed that PLLA has a substantial effect on the antimicrobial activity of PTFE only in the case of its combination with plasma modification or sputtered Ag nanolayer, resp. Ag deposited on PLLA applied to a PTFE nanofiber fabric exhibited excellent antimicrobial properties. Some antibacterial effect of a PLLA layer itself on PTFE nanotextile was observed for all tested bacterial strains.

#### 3.4.3. PFA

**Figure 15:**
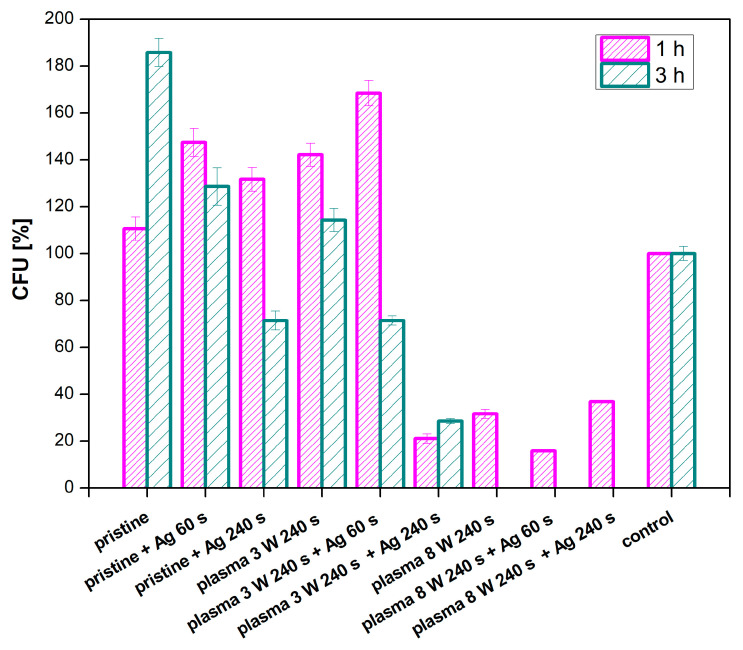
Results of antimicrobial tests for PFA using the bacterial strain *S. aureus* for samples, plasma-modified at 3 and 8 W for 240 s, and sputtered with Ag for 60 and 240 s.

**Figure 16:**
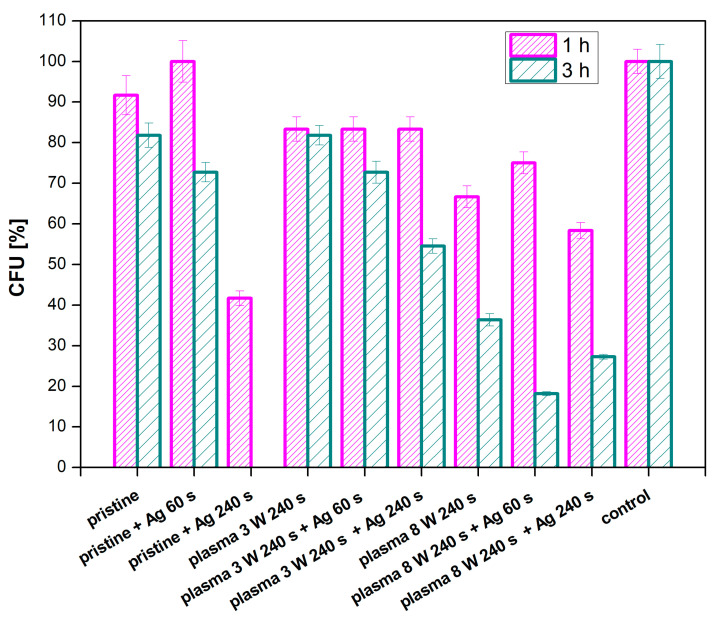
Results of antimicrobial tests for PFA using the bacterial strain *E. coli* for samples, plasma-modified at 3 and 8 W for 240 s and sputtered with Ag for 60 and 240 s.

In addition to PTFE, we were interested whether PFA could be used for the same application. PFA was also modified by plasma at 3 and 8 W with the exposure time of 240 s and combined with silver sputtering at deposition times of 60 and 240 s. The antibacterial properties of a PFA film were monitored against two bacterial strains of *S. aureus* (Figure 15) and *E. coli* (Figure 16).

**Figure 17:**
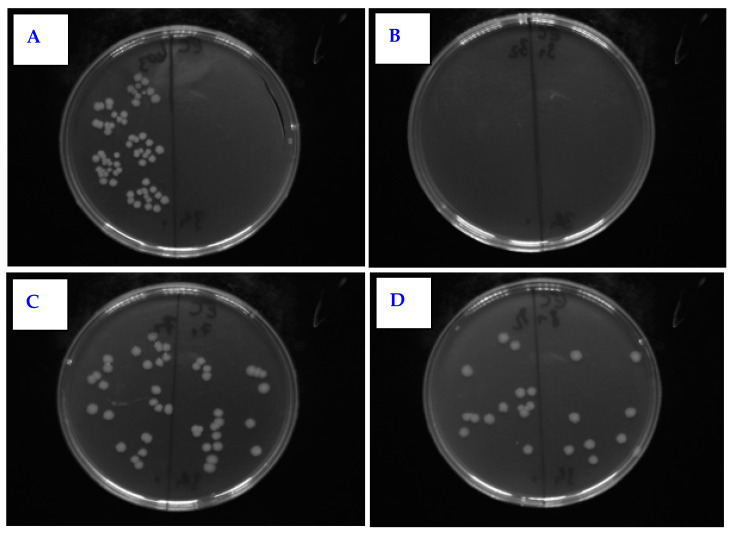
Selected photos of agar plates from antimicrobial tests for PFA against *E. coli* for samples: (**A**) control sample; (**B**) pristine + Ag 240 s; (**C**) plasma 8 W 240 s; (**D**) plasma 8 W 240 s + Ag 60 s.

As expected, a decrease in the number of grown bacteria was observed for all samples with increasing incubation time. Almost no antimicrobial activity appeared in the pristine PFA samples. When compared to the control sample, it is evident that the samples modified with plasma at the higher power (8 W) and the same samples in a combination with silver sputtering showed the best antibacterial effect. Under these conditions, no bacteria grew after 3 h of incubation with the samples. This indicates a direct effect of the plasma modification on the antimicrobial properties of PFA against the *S. aureus* strain. A lower number of CFU was detected on a sample modified at 3 W with sputtered silver, which confirms the higher efficiency of plasma modification at higher powers in a combination with silver. Another situation was observed for *E. coli*, for which the only effective combination was an unmodified sample of PFA sputtered for 240 s with silver (Figure 17). The plasma modification or the material itself had no effect on the antimicrobial activity of the PFA against *E. coli*.

If we summarize the antimicrobial effects of particular physico-chemical surface changes on the materials, two major effects take place. The first one is the antimicrobial effect of particular Ag coating and its influence on decrease of CFUs on most studied samples. The influence of Ag is surely connected with the form of isolated nanoclusters, which were confirmed to form by atomic force analysis, and the ability to release silver ions from nanostructured surface is the major factor influencing the antibacterial properties. From Ag nanoclusters, similarly as in the case of AgNPs, an oxidative release of Ag+ ions can occur after interaction with aqueous solution. Therefore, the antimicrobial mechanism of Ag nanoclusters can be explained by the Ag+ action involving Ag+ linking to the functional groups of bacterial proteins and enzymes, which induces inhibition of cell process as described in Reference [46]. Moreover, it has been previously shown in the ref. below that AgNPs are effective against bacteria in a similar way to Ag^+^, but in distinct effective concentrations (AgNPs and Ag^+^ at nanomolar and micromolar concentrations, respectively) [47]. Summarized, this means that Ag nanoclusters similarly to AgNPs acting as Ag+ could share the same mechanism of action of penetration into bacterial cells leading to condensation of DNA molecules, thus disabling replication leading to bacterial cell death [48]. Further, since Ag+ (possibly also released from Ag nanoclusters after contact with bacteria in aqueous solution) readily binds with the sulfhydryl group of bacterial proteins [49], it results in abolishing of multiple other proteins deleteriously diminishing bacterial cell propagation. A second important factor is the altered surface morphology, which has a great influence on PLLA coated samples, where a significant increase of surface roughness was observed, similar to honeycomb biopolymer pattern observed also on perfluorinated surfaces [14]. One of the major factors influencing the antimicrobial effectivity we may consider is the superhydrophobicity of material, which significantly contributes to antibacterial properties of material [50]. Although the values of contact angles remain relatively high for our samples, even after surface treatments, the contribution of wettability on antimicrobial activity may be considered only as minor factor in our case.

## 4. Conclusions

The surface properties of activated polymeric materials PTFE and PFA, their modification, and study of antibacterial properties were presented in detail in this article. The plasma-modified PTFE and PFA samples, samples with sputtered Ag nanostructures, and samples treated in a combination of these two techniques were performed. Surface wettability, surface morphology, chemical composition, and antimicrobial properties were investigated.

Analysis of the PTFE surface wettability showed that argon plasma causes a significant decrease in contact angles during 60 s treatment, and, with the increase in the length of this treatment, the wettability decreased again for both 3 and 8 W of plasma. However, at the modification time of 480 s, the PTFE surface wettability recovered to pristine sample values, which is an unusual phenomenon connected to the nanostructure of the textile, and it can be said that the plasma modification itself had no significant effect on wettability in the case of PTFE nanotextiles during longer exposure times. The difference in surface morphology between the two materials was analyzed from AFM images. At the same time, an increase in surface roughness with increasing plasma modification time at higher power was determined. The analysis of the chemical composition of the PTFE nanotextile showed the effect of the plasma on the subsequent binding of oxygen to the position of the fluorine in the surface layer of the polymer.

Studies of antimicrobial activity have shown that PTFE plasma-modified samples coated with PLLA and deposited with a thin layer of Ag have a strong antimicrobial effect against bacterial strains of *S. aureus* and *Proteus sp.*, and a slightly weaker antibacterial effect was observed against *E. coli*. Only PLLA-coated PTFE samples showed no significant resistance to these strains. A strong antibacterial effect was observed for the PFA material against the bacterial strain of *S. aureus*. Significant antibacterial effect for *E. coli* has been demonstrated from PTFE nanotextile plasma-treated for 240 s, coated with PLLA, and subsequently sputtered for 50 s of silver. Plasma modification of PFA alone did not affect antimicrobial activity, and the only effective combination was an unmodified sample of PFA sputtered for 240 s with silver.

## Figures and Tables

**Table 1:** Dependence of the thickness of a silver layer [nm] deposited on the PTFE and PFA surface.

**Ag deposition time [s]**	60	80	120	240	300
**Thickness [nm]**	3.1 ± 0.1	3.5 ± 0.1	6.4 ± 0.1	9.2 ± 0.2	9.8 ± 0.2

**Table 2:** Surface atomic concentrations of C, F, O, N elements determined by XPS analysis in pristine PTFE and PTFE treated by plasma at 3 and 8 W for 60, 120, and 240 s. The error of measurement did not exceed 2%.

Sample	Elemental Concentration (at. %)			
	C (1 s)	F (1 s)	O (1 s)	N (1 s)
Pristine PTFE	32.13	67.87	-	-
PTFE + plasma 3 W 60 s	36.00	59.13	4.55	0.32
PTFE + plasma 3 W 120 s	37.94	56.79	4.94	0.33
PTFE + plasma 3 W 240 s	40.46	50.80	8.38	0.36
PTFE + plasma 8 W 60 s	40.01	51.36	8.31	0.31
PTFE + plasma 8 W 120 s	40.14	50.93	8.51	0.43
PTFE + plasma 8 W 240 s	40.02	50.93	8.69	0.36

**Table 3:** Surface atomic concentrations of C, F, O, and N elements determined by XPS analysis in pristine PFA and PFA treated by plasma at 3 and 8 W for 60, 120, and 240 s. The error of measurement did not exceed 2%.

Sample	Elemental Concentration (at. %)			
	C (1s)	F (1s)	O (1s)	N (1s)
Pristine PFA	29.35	70.47	0.18	-
PFA + plasma 3 W 60 s	34.22	51.46	7.78	1.97
PFA + plasma 3 W 120 s	40.46	52.95	6.59	-
PFA + plasma 3 W 240 s	33.02	53.30	8.23	1.48
PFA + plasma 8 W 60 s	43.19	46.41	9.90	0.51
PFA + plasma 8 W 120 s	27.64	50.75	9.26	1.88

## Data Availability

Data is contained within the article or supplementary material.

## Supplementary Materials

The following are available online at https://www.mdpi.com/2079-4991/11/1/182/s1, Figure S1: XPS spectra of a pristine PTFE and plasma-treated PTFE sample at 8 W for 240 s.

## References

[1] Elbourne A., Crawford R.J., Ivanova E.P. (2017). Nano-structured antimicrobial surfaces: From nature to synthetic analogues. J. Colloid Interface Sci..

[2] Wang M., Tang T. (2019). Surface treatment strategies to combat implant-related infection from the beginning. J. Orthop. Transl..

[3] Olmo J.A.-D., Ruiz-Rubio L., Pérez-Alvarez L., Sáez-Martínez V., Vilas-Vilela J.L. (2020). Antibacterial coatings for improving the performance of biomaterials. Coatings.

[4] Adlhart C., Verran J., Azeveda N.F., Olmez H., Keinänen-Toivola M.M., Gouveia I., Melo L.F., Crijns F. (2018). Surface modifications for antimicrobial effects in the healthcare setting: A critical overview. J. Hosp. Infect..

[5] Huang K., Yang C., Huang S., Chen C., Lu Y., Lin Y. (2016). Recent advances in antimicrobial polymers: A mini-review. Int. J. Mol. Sci..

[6] Bazaka K., Jacob M.V., Chrzanowski W., Ostrikov K. (2015). Anti-bacterial surfaces: Natural agents, mechanisms of action, and plasma surface modification. RSC Adv..

[7] Hu Z., Chan W.L., Szeto Y.S. (2007). Nanocomposite of chitosan and silver oxide and its antibacterial property. J. Appl. Polym. Sci..

[8] Kumar N., Desagani D., Chandran G., Ghosh N.N., Karthikeyan G., Waigaonkar S. (2017). Biocompatible agarose-chitosan coated silver nanoparticle composite for soft tissue engineering applications. Artif. Cells Nanomed. Biotechnol..

[9] Slepička P., Slepičková Kasálková N., Stránská E., Bačáková L., Švorčík V. (2013). Surface characterization of plasma treated polymers for applications as biocompatible carriers. Express Polym. Lett..

[10] Slepička P., Peterková L., Rimpelová S., Pinkner A., Slepičková Kasálková N., Kolská Z., Ruml T., Švorčík V. (2016). Plasma activated perfluoroethylenepropylene for cytocompatibility enhancement. Polym. Degrad. Stab..

[11] Peterková L., Rimpelová S., Slepička P., Slepičková Kasálková N., Veselý M., Švorčík V., Ruml T. (2016). Bioinert fluorinated ethylene-propylene copolymer modified for keratinocyte adhesion. FEBS J..

[12] Slepička P., Neznalová K., Fajstavr D., Švorčík V. (2020). Nanostructuring of honeycomb-like polystyrene with excimer laser. Prog. Org. Coat..

[13] Neznalová K., Sajdl P., Švorčík V., Slepička P. (2020). Cellulose acetate honeycomb-like pattern created by improved phase separation. Express Polym. Lett..

[14] Neznalová K., Fajstavr D., Rimpelová S., Slepičková Kasálková N., Kolská Z., Švorčík V., Slepička P. (2020). Honeycomb-patterned poly(L-lactic) acid on plasma-activated FEP as cell culture scaffold. Polym. Degrad. Stab..

[15] Fajstavrová K., Rimpelová S., Fajstavr D., Švorčík V., Slepička P. (2020). PLLA honeycomb-like pattern on fluorinated ethylene propylene as a substrate for fibroblast growth. Polymers.

[16] Feng S.S., Zhong Z.X., Wang Y., Xing W.H., Drioli E. (2018). Progress and perspectives in PTFE membrane: Preparation, modification, and applications. J. Membr. Sci..

[17] Kolská Z., Řezníčková A., Hnatowicz V., Švorčík V. (2012). PTFE surface modification by Ar plasma and its characterization. Vacuum.

[18] Zhang S., Liang X., Gadd G.M., Zhao Q. (2019). Advanced titanium dioxide-polytetrafluorethylene (TiO_2_-PTFE) nanocomposite coatings on stainless steel surfaces with antibacterial and anti-corrosion properties. App. Surf. Sci..

[19] Yoon H.J., Kim S.E., Kwon Y.K., Kim E.J., Lee J.C., Lee Y.S. (2012). Synthesis of silver nanostructures on polytetrafluoroethylene (PTFE) using electron beam irradiation for antimicrobacterial effect. J. Ind. Eng. Chem..

[20] Guo R., Yin G., Sha X., Zhao Q., Wei L., Wamg H. (2015). The significant adhesion enhancement of Ag–polytetrafluoroethylene antibacterial coatings by using of molecular bridge. Appl. Surf. Sci..

[21] Akinci A., Cobanoglu E. (2009). Coating of Al mould surfaces with polytetrafluoroethylene (PTFE), fluorinated ethylene propylene (FEP), perfluoroalkoxy (PFA) and ethylene-tetrafluoroethylene (ETFE). e-Polymers.

[22] Sprang N., Theirich D., Engemann J. (1998). Surface modification of fluoropolymers by microwave plasmas: FTIR investigations. Surf. Coat. Technol..

[23] Zhaia M., Gong Y., Chena X., Xiao T., Zhang G., Xu L., Li H. (2017). Mass-producible hydrophobic perfluoroalkoxy/nano-silver coatings bysuspensionflame spraying for antifouling and dragreduction applications. Surf. Coat. Technol..

[24] Slepičková Kasálková N., Slepička P., Kolská Z., Hodačová P., Kučková Š., Švorčík V. (2014). Grafting of bovine serum albumin proteins on plasma-modified polymers for potential application in tissue engineering. Nanoscale Res. Lett..

[25] Neděla O., Slepička P., Sajdl P., Veselý M., Švorčík V. (2017). Surface analysis of ripple pattern on PS and PEN induced with ring-shaped mask due to KrF laser treatment. Surf. Interface Anal..

[26] Slepicka P., Siegel J., Lyutakov O., Slepickova Kasalkova N., Kolska Z., Bacakova L., Svorcik V. (2018). Polymer nanostructures for bioapplications induced by laser treatment. Biotechnol. Adv..

[27] Liu F., Hashim N.A., Liu Y., Mhgareg Abed M.R., Li K. (2011). Progress in the production and modification of PVDF membranes. J. Membr. Sci..

[28] Liu C., Wu L., Zhang C., Chen W., Luo S. (2018). Surface hydrophilic modification of PVDF membranes by trace amounts of tannin and polyethyleneimine. Appl. Surf. Sci..

[29] Lim S.J., Shin I.H. (2020). Graft copolymerization of GMA and EDMA on PVDF to hydrophilic surface modification by electron beam irradiation. Nucl. Eng. Technol..

[30] Yavari M., Okamoto Y., Lin H. (2018). The role of halogens in polychlorotrifluoroethylene (PCTFE) in membrane gas separations. J. Membr. Sci..

[31] Ebnesajjad S. (2017). Introduction to fluoropolymers. Applied Plastics Engineering Handbook.

[32] Brobbey K.J., Haapanen J., Makela J.M., Gunell M., Eerola E., Rosqvist E., Peltonen J., Saarinen J.J., Tuominen M., Toivakka M. (2019). Effect of plasma coating on antibacterial activity of silver nanoparticles. Thin Solid Film..

[33] Okubo M., Tahara M., Saeki N., Yamamoto T. (2008). Surface modification of fluorocarbon polymer films for improved adhesion using atmospheric-pressure nonthermal plasma graft-polymerization. Thin Solid Film..

[34] Tripathi J., Sharma S., Tripathi S., Bisen R., Agrawal L. (2017). Modifications in optical and structural properties of PMMA/PCTFE blend films as a function of PCTFE concentration. Mater. Chem. Phys..

[35] Shan D., Grhard E., Zhang C., Tierney J.W., Xie D., Liu Z., Yang J. (2018). Polymeric biomaterials for biophotonic applications. Bioact. Mater..

[36] Korzinskas T., Jung O., Smeets R., Stojanovic S., Najman S., Glenske K., Barbeck M. (2018). In vivo analysis of the biocompatibility and macrophage response of a non-resorbable PTFE membrane for guided bone regeneration. Int. J. Mol. Sci..

[37] Abbasian M., Massoumi B., Mohammad-Rezaei R., Samadian H., Jaymand B. (2019). Scaffolding polymeric biomaterials: Are naturally occurring biological macromolecules more appropriate for tissue engineering?. Int. J. Biol. Macromol..

[38] Faustino C.M.C., Lemos S.M.C., Monge N., Ribeiro I.A.C. (2020). A scope at antifouling strategies to prevent catheter-associated infections. Adv. Colloid Interface Sci..

[39] Cardoso V.F., Correia D.M., Ribeiro C., Fernandes M.M., Lanceros-Méndez S. (2018). Fluorinated polymers as smart materials for advanced biomedical applications. Polymers.

[40] Dhandayuthapani B., Sakthi Kumar D. (2016). Biomaterials for Biomedical Applications.

[41] Gomes M.F., Amorim J.B., Jianassi L.C., Castillo Salgado M.A. (2018). Biomaterials for Tissue Engineering Applications in Diabetes Mellitus.

[42] Shao H.J., Chen C.S., Lee I.C., Wang J.H., Young T.H. (2009). Designing a three-dimensional expanded polytetrafluoroethylene-poly(lactic-co-glycolic acid) scaffold for tissue engineering. Artif. Organs.

[43] Banoriya D., Purohit R., Dwivedi R.K. (2017). Advanced application of polymer based biomaterials. Mater. Today Proc..

[44] Lv J., He B., Yu J., Wang Y., Wang C., Zhang S., Wang H., Hu J., Zhang Q., Cheng Y. (2018). Fluoropolymers for intracellular and in vivo protein delivery. Biomaterials.

[45] Tokiwa Y., Calabia B.P. (2006). Biodegradability and biodegradation of poly(lactide). Appl. Microbiol. Biotechnol..

[46] Sohrabnezhad S., Pourahmad A., Mehdipour Moghaddam M.J., Sadeghi A. (2015). Study of Antibacterial Activity of Ag and Ag_2_CO_3_ Nanoparticles Stabilized Over Montmorillonite. Spectrochim. Acta Part A.

[47] Lok C.N., Ho C.M., Chen R., He Q.Y., Yu W.Y., Sun H., Tam P.K., Chiu J.F., Che C.M. (2006). Proteomic analysis of the mode of antibacterial action of silver nanoparticles. J. Proteome Res..

[48] Feng Q.L., Wu J., Chen G.Q., Cui F.Z., Kim T.N., Kim J.Q. (2000). A mechanistic study of the antibacterial effect of silver ions on Escherichia coli and Staphylococcus aureus. J. Biomed. Mater. Res..

[49] Liau S.Y., Read D.C., Pugh W.J., Furr J.R., Russell A.D. (1997). Interaction of silver nitrate with readily identifiable groups: Relationship to the antibacterial action of silver ions. Lett. Appl. Microbiol..

[50] Zhang X., Wangband L., Levanen E. (2013). Superhydrophobic surfaces for the reduction of bacterial adhesion. RSC Adv..

