# Supplementary material for: Antibacterial Properties of Plasma-Activated Perfluorinated Substrates with Silver Nanoclusters Deposition
Source: Nanomaterials (Basel). 2021 Jan 13;11(1):182. doi: 10.3390/nano11010182 (PMC7828452; doi:10.3390/nano11010182)
Supplement: Supplementary file 1 [file nanomaterials-11-00182-s001.pdf]

nanomaterials

Supplementary Material

Antibacterial Properties of Plasma-Activated Perfluorinated Substrates with Silver Nanoclusters Deposition

Petr Slepička [1,*], Silvie Rimpelová [2,*], Nikola Slepičková Kasálková [1], Dominik Fajstavr [1], Petr Sajdl [3], Zdeňka Kolská [4] and Václav Švorčík [1]

[1] Department of Solid State Engineering, University of Chemistry and Technology Prague, 166 28 Prague, Czech Republic; nikola.kasalkova@vscht.cz (N.S.K.); dominik.fajstavr@vscht.cz (D.F.) vaclav.svorcik@vscht.cz (V.Š)
[2] Department of Biochemistry and Microbiology, University of Chemistry and Technology Prague, 166 28 Prague, Czech Republic
[3] Department of Power Engineering, University of Chemistry and Technology Prague, 166 28 Prague, Czech Republic; petr.sajdl@vscht.cz
[4] Faculty of Science, J. E. Purkyně University in Ústí nad Labem, 400 96 Ústí nad Labem, Czech Republic; zdenka.kolska@ujep.cz
* Correspondence: petr.slepicka@vscht.cz (P.S.); silvie.rimpelova@vscht.cz (S.R.)

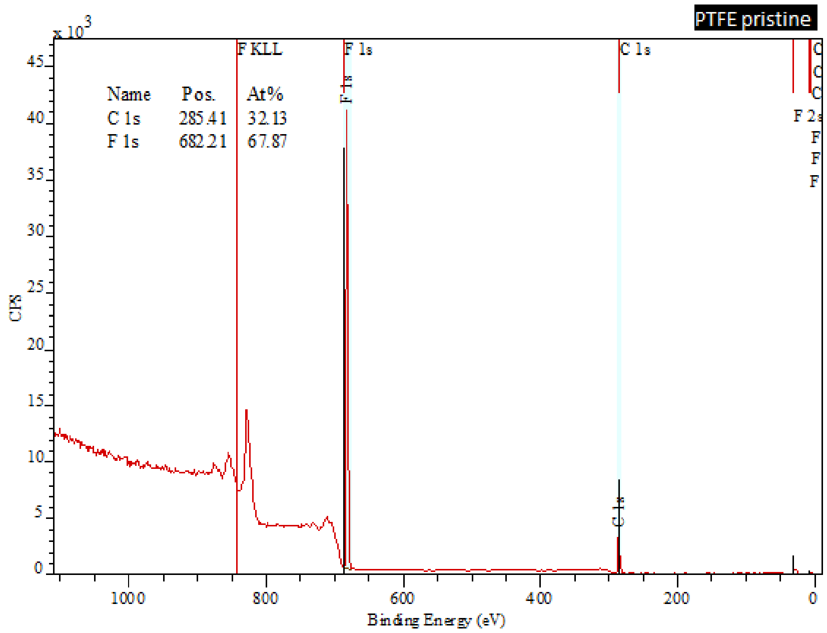

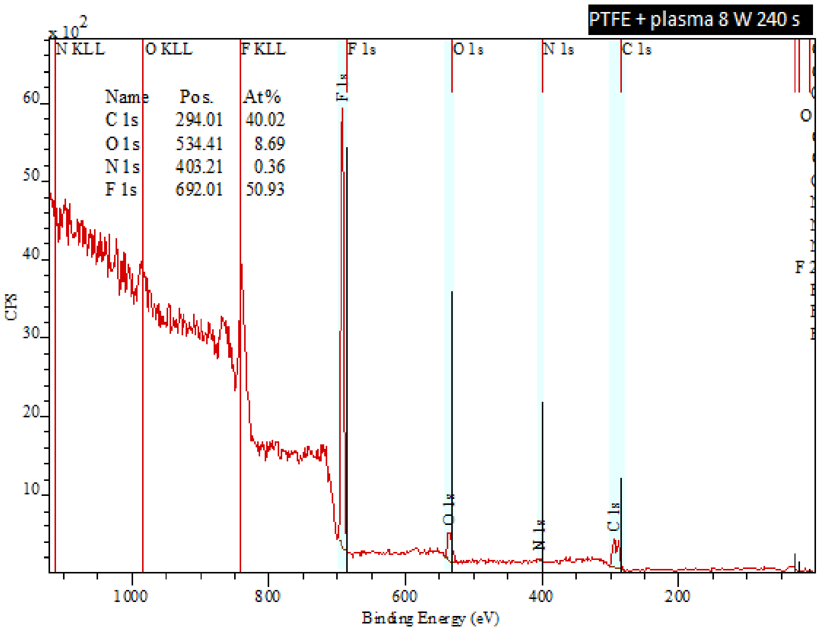

Figure S1. XPS spectra of a pristine PTFE and plasma-treated PTFE sample at 8 W for 240 s.
